# Injecting Drug Users and Their Health Seeking Behavior: A Cross-Sectional Study in Dhaka, Bangladesh

**DOI:** 10.1155/2015/756579

**Published:** 2015-01-27

**Authors:** Sheikh Mohammed Shariful Islam, Tuhin Biswas, Faiz Ahmed Bhuiyan, Md. Serajul Islam, Mohammad Mizanur Rahman, Hurun Nessa

**Affiliations:** ^1^Center for Control of Chronic Diseases (CCCD), International Center for Diarrhoeal Disease Research, Bangladesh (icddr,b), 68 Shaheed Tajuddin Ahmed Sarani, Mohakhali, Dhaka 1212, Bangladesh; ^2^Center for International Health (CIH), Ludwig Maximilians University (LMU), 7 Leopoldstraße, 80802 Munich, Germany; ^3^Research Administration, International Center for Diarrhoeal Disease Research, Bangladesh (icddr,b), 68 Shaheed Tajuddin Ahmed Sarani, Mohakhali, Dhaka 1212, Bangladesh; ^4^Department of Community Medicine, Ad-din Sakina Medical College, Jessore 7400, Bangladesh; ^5^Center for Child and Adolescent Health, International Center for Diarrhoeal Disease Research, Bangladesh (icddr,b), 68 Shaheed Tajuddin Ahmed Sarani, Mohakhali, Dhaka 1212, Bangladesh; ^6^Staff Clinic, Ha-Meem Group, Tejgaon, Dhaka 1212, Bangladesh

## Abstract

*Introduction and Aim.* Injecting drug users (IDUs) are amongst the most vulnerable people to acquisition of HIV/AIDS. This study aims to collect information on IDUs and their health seeking behavior in Bangladesh. *Design and Methods.* A cross-sectional study was conducted among 120 IDUs attending a drug rehabilitation center in Dhaka, Bangladesh. Data were collected on sociodemographics, drug use, health seeking behavior, knowledge of injecting drugs, and sexual behavior. *Results.* The mean ± SD and median (IQR) age of the participants were 32.5 ± 21.3 and 33 (27–38) years, respectively, with only 9.2% females. Injection buprenorphine was the drug of choice for 40% of participants, and 58% of the participants first started drug use with smoking cannabis. 73.3% of participants shared needles sometimes and 57.5% were willing to use the needle exchange programs. 60% of the participants had no knowledge about the diseases spread by injection. Condom use during the last intercourse with regular partners was 11.7% and with any partners 15.8%. *Conclusion.* IDUs in Bangladesh are a high-risk group for HIV/AIDS due to lack of knowledge and risky behaviors. Education and interventions specifically aimed at IDUs are needed, because traditional education may not reach IDUs or influence their behavior.

## 1. Introduction

Drug abuse is an alarming and complex problem in Bangladesh where there are an estimated 1.7 million drug abusers and the number has been increasing greatly over recent years [1]. According to a research in Bangladesh, the amount of money drug abusers spent per year is US$707–1,135 per person which was much higher than the per-capita income of Bangladeshi people (US$380 in 2001) [1]. The study projected that the total amount spent by 1.7 million drug abusers in Bangladesh would be US$1.2–1.9 billion. Moreover, the actual cost of drug abuse would be far more if we include other related social and economic impacts. Drug users and injecting drug users (IDUs) are people who use or inject illegal drugs despite negative consequences to one's health. Drug use has played a major role in the spread of HIV/AIDS in many countries. The high level of HIV infection among IDUs is a major health problem of international concern [2]. Drug users also function as a “bridge” for HIV transmission to the heterosexual population and to children through perinatal transmission [3]. Bangladesh is predominantly a Muslim country with over 150 million people, located between India and Myanmar, and a low prevalence country for HIV/AIDS. The estimated number of IDUs in Bangladesh was 20000–40000 in 2008 and the numbers are increasing rapidly [4, 5]. The capital city Dhaka has more than 12 million people. A previous study in Bangladesh reported HIV epidemic concentrated among IDUs in Dhaka city [6].

In the past few years, some 10–20 percent of the drug users have become new injectors. In the southeast of the country, 60% of all IDUs have only started injecting within the last two years [7]. The prevalence of HIV in Bangladesh is less than 0.1% in the general population and has remained less than 1% over the years including most at risk population such as clients of sex workers and IDUs [8]. However, studies showed that although Bangladesh is a low prevalence country for HIV/AIDS, all the factors that may allow rapid spread of an AIDS epidemic are present in this country. These include high-risk behavior, lack of awareness, very mobile populations, and being surrounded by countries that have a higher prevalence of HIV. According to the latest Serological Surveillance (Round 9, 2011) of Bangladesh, the HIV prevalence among drug users, male and female sex workers, men who have sex with men (MSM), and transgender or hijras was 0.7% [4].

The Round 9 Surveillance tested 7,529 drug users (IDUs, heroin smokers, and the combined group of IDUs and heroin smokers) from 30 different cities in Bangladesh. Overall HIV prevalence was 1.2%, with low rates found in drug users from five cities. In Dhaka the prevalence of HIV among IDUs was 5.3%. Though active syphilis rates among IDUs declined significantly over time in Dhaka, there were no significant changes in the other cities where trend analysis was possible. Hepatitis C virus (HCV) was present in over 50% of IDUs in six of the cities. However, the highest prevalence of HCV was found among IDUs in several cities, with 95.7% as the highest in a northwestern city [4, 9]. Another study among IDUs in Bangladesh reported the prevalence of HIV 5.6% [10]. However, treatment for drug dependence and needle exchange programs (NEPs) are virtually the only services available to IDUs in Bangladesh, mostly through NGOs and with little support from the government. Opioid substitution therapy and access to antiretroviral treatment for IDUs were generally not available in the country [11].

There is growing recognition that healthcare seeking behaviors and local knowledge need to be considered in programs and interventions to promote health in a variety of contexts [7]. Previous studies in Bangladesh have reported the prevalence and different programs directed towards the IDUs. Information regarding personal profile of IDUs and their health seeking behavior is needed to develop prevention strategies and policy recommendation to combat HIV/AIDS in this risky group of population. However, there is limited data on IDUs and their health seeking behavior in Bangladesh. We, therefore, conducted this study to identify the personal profile of injecting drug users (IDUs) in Dhaka city and their health seeking behavior.

## 2. Materials and Methods

### 2.1. Study Design, Place, and Population

We conducted a cross-sectional study between March and September 2005 among 120 IDUs attending the Society for Community-Health Rehabilitation Education and Awareness (CREA), a drug addiction treatment and rehabilitation center at Mohammadpur, Dhaka. Participants were selected purposely from newly admitted cases attending a counseling session at the center and referred by attending physician/counselor. The inclusion criteria were as follows: participants who used injection of one or more drugs (excluding insulin or other prescribed medications for any illness) within the last 3 months prior to the interview, admission within the last 30 days, no history of severe mental disorders, and ability to provide written informed consent.

### 2.2. Data Collection Tools

Data were collected through face-to-face interviews using a structured questionnaire in Bengali. The questionnaire was developed by a team of epidemiologists, physicians, psychologists, and social scientists and pretested among 20 IDUs attending the outpatient department at a drug addiction treatment center in Tejgaon, Dhaka. During the pretest phase we considered if the questions were too sensitive to answer, level of difficulty, and privacy of the respondents. After pretest, the questionnaire was modified and used for data collection in this study.

### 2.3. Ethics

Before data collection, permission was obtained from the head of the drug addiction treatment center and attending physicians and counselors. All participants in the study were informed of the objectives of the study and that they were free to participate by their own will and that their opinion or withdrawal will have no consequences on their treatments. Written informed consent was obtained from all the participants prior to the interview. The study protocol was approved by the Institutional Review Board of the National Institute of Preventive and Social Medicine (NIPSOM).

### 2.4. Variables, Data Entry, and Analysis

Information on age, sex, religion, living alone or with family, marital status, number of dependencies in the family, education, occupation, source of income, and monthly average income by the respondent (self-income) and types of drug used and duration was collected. Health seeking behavior in terms of primary point of care, reason for delay or not taking treatment earlier, information about needle sharing, reasons for needle sharing, and information regarding needle exchange program (NEP) were collected. Sharing means using someone else's equipment, which has already been used, or someone using the respondents' equipment, regardless of whether both the participant and the user were present at the time. Injecting equipment included needles, syringes, filters, spoons and cookers, and washouts. Sharing of needles and equipment was classified as “always” = shared during every use, “sometimes” = shared anytime, and “never” = never shared. NEP refers to organized needle exchange programs available to IDUs through NGOs and at no cost. We also collected information on level of knowledge about the consequences of the diseases transmitted by injecting drugs and about the sexual behavior of the respondents such as sex with regular partners, sex during the last three months, condom use during the last sexual intercourse, and condom use with any partners. Regular partner refers to having sexual intercourse with a single partner on a regular basis, such as a wife or a fixed girlfriend.

All data were prospectively recorded on case report forms. Data that were missing, inconsistent, or both were obtained or clarified by direct communication of the data collectors. Data entry and analysis was performed using Microsoft Excel. Data were presented as frequency (*n*) and percentage (%). Mean and standard deviation were calculated for normal distribution and median and interquartile range (IQR) were reported for nonnormal distribution data.

## 3. Results

Table 1 shows the sociodemographic characteristics of study participants. 120 IDUs participated in this study with a mean ± SD age of 32.5 ± 21.3 years. The age range was from 13 to 62 years. Almost half (48%) of the participants were in the age group 30–39 years, followed by 21.7% aged 40 years or more, and the rest were in other age groups. 91% of the study participants were male. A great majority of the IDUs were Muslims (76.7%), living alone (57.5%), and unmarried (45.0%) and had no dependent person to take care of (40.8%). More than two-thirds of the respondents had no education or only completed primary level of education. About half of the participants (55%) were unemployed and 49.2% mentioned self-income as the only sources of income. The mean ± SD monthly income of the participants was 10450 ± 7300 Bangladeshi Takas (BDT) (1 US$ = 78.9 BDT).

Drug use of the participants is presented in Table 2. The main drug used or the drug of choice was injection buprenorphine (40.0), followed by other injections or mixed form of injections (25.8%), injection pethidine (18.3%), and injection sedatives (15.8%). Almost half of the participants (50.8%) mentioned smoking cannabis (ganja or marijuana) as the first drug used and 18.3% started with a cough-syrup (Phensedyl). A great majority of the participants (51.7%) started taking first drug during the age of 16–24 years and less than 10% initiated drug use after 44 years. However, a greater number of participants (30.8%) mentioned injecting first drug during the age of 25–34 years. The average duration of taking injectable drug was 15.4 ± 2.3 months.

Table 3 presents the health seeking behavior of the respondents. About one-third (33.3%) of the participants reported local MBBS (Bachelor of Medicine and Bachelor of Surgery) doctors or registered physicians as primary point of care, followed by private medical centre (19.2%), government health centre (10.8%), local pharmacy (10.0%), and paramedics, Ayurvedic, homeopathics, quacks, or traditional healers (8.3%). About 18.3% of the participants reported not seeking healthcare before. During the interview, the majority of the respondents (40.0%) mentioned that for drug addiction problem they did not know where to go for treatment, 30% thought that treatment was too expensive, 17.5% thought that treatment was not effective, and only 15% mentioned other causes. Needles and syringes sharing was a common practice for 40% of participants, while 33.3% of participants reported sharing needles/syringes sometimes. Among all the respondents, only 4% of participants reported practicing needle sharing always. Participants reported needle sharing because it was cheap (10%), was convenient (18.3%), and was considered as a norm (15%). More than half of the participants (57.5%) were willing to use the needle exchange programs (NEPs), while 30% mentioned not being willing to use NEPs. Costs or loss of income (12.5%), fear of police (5.8%), and social problems (7.5%) were reasons for not being willing to use the NEPs.

Respondents' knowledge about the diseases spread by injecting drug use is presented in Table 4. The majority of the respondents (60.0%) had no knowledge about the diseases spread through injections and needle sharing. Only 17.5% of participants could mention HIV/AIDS, 6.7% syphilis, 5.8% hepatitis B or C, and 2.5% other blood-borne diseases and 67.5% could not mention anything. Regarding knowledge about protection, 29.2% of participants mentioned not injecting any more drugs, 34.2% mentioned cleaning syringe/needle, 10% mentioned using condoms, and 5% reported that taking medicine can protect them from harms. When asked about the consequences of drug use, the majority of the participants mentioned the adverse effects of injecting drug use on their health, occupation, and family (Table 4).

Table 5 presents the sexual behavior of the participants. A great majority of the participants had sex with regular partners. However, only 11.7% of participants mentioned condom use during the last sexual intercourse. When asked about condom use with any sex partners, only 15.8% of the participants reported using condoms.

## 4. Discussion

Results of our study showed that IDUs in Bangladesh are most vulnerable to acquisition and spread of HIV/AIDS among general population due to lack of awareness and knowledge about HIV/AIDS and practicing risky behaviors such as high level of needle/syringe sharing and unprotected sex. The majority of the IDUs in this study were young and middle-aged men. This may be due to the fact that females prefer taking injections and treatment at home instead of drug rehabilitation centers due to social stigma [6, 12]. The recent increase in shift to injection might result from easy availability of injecting drugs, relatively cheaper price compared to other drugs, and better surveillance and reporting [12, 13]. A previous study in Bangladesh reported 78% of IDUs continued stably exchanging needles and syringes, but the reported rate of condom use in commercial sex remained low, improving from only 7.8% to 17.7% [14, 15]. Other studies in Bangladesh among IDUs also reported risky sexual behaviors among IDUs compared to non-IDUs [16, 17].

Another study among 505 “drug addicts” in Dhaka showed those who were HIV positive (3.7%) were mainly IDUs, and all of them shared needles. The majority of the participants (73.3%) of our study also reported sharing needles. Those drug users with HIV were also more likely to report unprotected sex (76.4%), having multiple sex partners (87.1%), and the presence of STIs (64.2%) [14]. These reports are consistent with our findings.

The results of our study showed low awareness about the consequences of drug use and about HIV, which is consistent with previous studies [18–20]. In general, most IDUs have heard of HIV/AIDS but they are unaware of how it spreads by injecting drugs. A marker for prevalent injection drug use is the prevalence of hepatitis C [16]. In Dhaka the prevalence of hepatitis C declined significantly over the years, which showed that safer injection practices are being adopted [4]. Early intervention efforts in Bangladesh, including extensive NEPs, are estimated to have reduced infections among IDUs and also contributed to decrease of the transmission to other populations [21]. However, these studies have been conducted among selected population groups and intervention subjects and do not represent the general IDUs in the country.

It is reported that the HIV epidemics of several other countries in Asia started off in IDUs and then spread via needle sharing and unprotected sex with the sex workers and thereafter to the general population [22]. In China, nearly half of all people infected with HIV are believed to have become infected through injecting drug use. There is often an overlap between communities of IDUs and communities of sex workers in many parts of Asia, as those who sell sex may do it to fund a drug habit, or they may have been involved in sex work first before turning to drug use [23]. In Bangladesh, around 75% of IDUs reported having unprotected sex in their last sexual encounter with a commercial sex worker [15]. Previous reports from Bangladesh showed that female IDUs are particularly vulnerable, as most of them reported selling sex to support their addiction and depended on their male partners to buy their drugs and shared needles with them [24]. Another study in Bangladesh reported that among female IDUs 61% reported selling sex [6]. In our study, of the 11 female participants, none reported any self-income or occupation as sex worker.

HIV can spread extremely rapidly among IDUs sharing needles, and IDUs provide a “bridge” for HIV transmission to their sexual partners and children. One approach has been to attempt to reduce drug use by strengthening drug interdiction and enforcement policies [25]. However, such policies are not necessarily effective in controlling the drug supply; they may simply reroute drug trade elsewhere, and they may indirectly result in raising risky behavior that spreads HIV.

The Narcotics Control Act (1990) of Bangladesh made drug use a criminal offense and drug users criminals and called for mandatory treatment of drug users. The act gave law enforcement agents control over drug sales and use and provision for harassment of drug sellers and users. However, the National AIDS Policy and the National HIV/AIDS Strategic Plan incorporated harm reduction services for IDUs in its strategic plan and law reform remains an urgent need in order to facilitate intervention activities with drug users.

There is a need to understand the barriers as well as the facilitators for NEPs among IDUs in order to improve health seeking behavior and awareness. Our data showed that a great majority of the participants did not know where to seek treatment, considered treatment too expensive, and were willing to use NEPs. Thus, efforts to promote NEPs through government funded programs and creating awareness among the IDUs in Bangladesh are needed. The keys to reducing the rapid spread of HIV/AIDS among IDUs are, first, recognition of injecting drug use as a potential problem and, second, early intervention that includes harm reduction and drug treatment [26, 27]. Evidence shows that IDUs do change their risky injecting behavior as a result of harm reduction programs and sometimes without large-scale intervention efforts [2, 27]. The two important components for harm reduction programs are education and provision of the means with which to change behavior. The latter may include sterile needles (through either NEPs or availability in pharmacies without a prescription), bleach, and/or drug rehabilitation programs. Interventions to prevent sexual transmission from IDUs to their partners through increased condom use have met with only limited success but are vital for preventing the spread of HIV out of the injecting group and into the general population [28].

The WHO defined a comprehensive package of nine interventions for IDUs, of which the following four have evidence for effectiveness in reducing HIV incidence: needle and syringe programs (NSP), medication-assisted therapy (MAT), antiretroviral therapy (ART), and HIV counseling and testing (HCT) [2]. At least 60% coverage is likely to be required to reduce HIV incidence. Evidence from LMIC contexts suggests that NSP and MAT can reduce high-risk injecting behavior, HCT can reduce risky sexual behavior, and ART can plausibly have preventive benefit among IDUs for onward parenteral transmission with clearer evidence that antiretroviral therapy (ARV) can prevent onward sexual transmission. Modeling analysis suggests that, compared with current low coverage, a scale-up of these four interventions in combination would be a beneficial and cost-effective approach [2].

Despite several risk factors, the HIV/AIDS prevalence in Bangladesh remains considerably low, which might be due to the early interventions by the government and several NGOs, targeted at most at risk populations. Previous studies in Nepal and Indonesia have reported that HIV prevalence among IDUs remained low for several years but then increased to over 45% [2]. These studies also highlight the importance of early interventions in the epidemic. However, currently the coverage of interventions for IDUs is inadequate in Bangladesh [29]. While the response to date has reduced the level of HIV transmission and ensured many people living with HIV (PLHIV) were under NEP coverage and received treatment [30], sufficient justification exists on human rights and public-health grounds for scaling up evidence-based prevention of HIV infection among IDUs. Social and structural changes for a combination intervention approach for HIV/AIDS prevention are essential. Bangladesh still has a rare window of opportunity to prevent significant HIV epidemic by providing prevention service to those at most risk of HIV infection including IDUs.

### 4.1. Limitations of the Study

Our study had several limitations. First, the study participants were recruited from a single drug addiction treatment site in Dhaka city. This group is likely to have better health seeking behaviors than those who do not seek treatment in a rehabilitation center. Therefore, the results may not generalize to other IDUs in Bangladesh. Second, the study participants were selected purposively. Therefore, chances of selection bias could not be ruled out. Third, our sample size was limited to 120 participants with a small number of female IDUs to compare the gender differences. Finally, due to limited resources we were not able to conduct statistical tests to determine the predictors of injecting drug use and health seeking behavior. Further studies with qualitative methods and evaluation of combined interventions and cost-effectiveness of the interventions are recommended to identify the best solution for Bangladesh.

## 5. Conclusion

Results of our study show that IDUs in Bangladesh have several risky behavior practices and are at high risk of developing HIV. Awareness building, education, and interventions specifically aimed at IDUs are needed, because programs targeted at the general population may not reach IDUs or influence their behavior. Scaling up and strengthening the use of condoms and NEPs might help to reduce harm in this vulnerable population group in Bangladesh and similar other developing countries.

## Figures and Tables

**Table 1:** Sociodemographic information of the respondents (*n* = 120).

Variables	Number (*n*)	Percentage (%)
Age group in years		
10–19	13	10.8
20–29	23	19.2
30–39	58	48.3
40 or more	26	21.7

Sex		
Male	109	90.8
Female	11	9.2

Religion		
Muslim	92	76.7
Hindu	17	14.2
Christians and others	11	9.2

Living status		
Alone	69	57.5
With family	51	42.5

Marital status		
Married	47	39.2
Unmarried	54	45.0
Divorcee/separated	19	15.8

Number of dependent persons		
None	49	40.8
1-2	32	26.6
3-4	25	20.8
More than 4	14	11.6

Education		
None	39	32.5
Primary (up to grade 5)	46	38.3
Secondary (up to grade 12)	23	19.2
Graduate/masters (bachelor's degree and above)	12	10.0

Occupation		
Unemployed	66	55.0
Student	12	10.0
Govt. service	5	4.2
Business	28	23.3
Others	9	8.0

Source of income		
Self	59	49.2
Parents or spouse	40	33.3
Others/no income	21	17.5

Average monthly self-income (in Taka)		
Mean ± SD (10450 ± 7330)		
<5000	26	21.7
5000–10000	42	35.0
10001–15000	30	25.0
15001 and above	22	18.3

**Table 2:** Distribution of respondents by drug use (*n* = 120).

Variables	Number (*n*)	Percentage (%)
Current drug of choice		
Injection pethidine	22	18.3
Injection buprenorphine	48	40.0
Injection sedatives	19	15.8
Injection (others)/mixed injections	31	25.8

Names of first drug use		
Injection pethidine	10	8.3
Injection buprenorphine	6	5.0
Injection sedatives	8	6.7
Injection (others)	9	7.5
Phensedyl	22	18.3
Ganja	61	50.8
Heroin	3	2.5
Others (noninjecting)	1	0.8

Age of taking first drug (years)		
<16	19	15.8
16–24	62	51.7
25–34	18	15
35–44	10	8.3
45–54	7	5.8
>55	4	3.3

Age of first injecting drug (years)		
<16	7	5.8
16–24	17	14.2
25–34	37	30.8
35–44	26	21.7
45–54	10	8.3
>55	3	2.5

Duration of injecting drug use		
<6 months	30	25.0
6 months–<1 year	21	17.5
1–3 years	22	18.3
3–5 years	27	22.5
More than 5 years	20	16.7

**Table 3:** Distribution of respondents by health seeking behavior (*n* = 120).

Variables	Number (*n*)	Percentage (%)
Primary point of care		
Govt. center	13	10.8
Private center	23	19.2
Local MBBS doctor	40	33.3
Local pharmacy	12	10.0
Paramedics/quacks/traditional	10	8.3
None	22	18.3

Reason for delay or not taking treatment earlier		
Don't know where to go	48	40.0
Treatment too expensive	36	30.0
Treatment not effective	21	17.5
Other causes	15	12.5

Needle sharing		
Always	48	40.0
Never	32	26.7
Sometimes	40	33.3

Reason for needle sharing (*n* = 88)		
Cheap	12	10
Convenient	22	18.3
No problem known	24	20
Norm	18	15
Don't know	12	10

Willing to use NEP		
Yes	69	57.5
No	36	30.0
Don't know	15	12.5

Reason for not being willing to use NEP (*n* = 36)		
Costs or loss of income	15	12.5
Fear of police	7	5.8
Social problems	9	7.5
Don't know	5	4.2

**Table 4:** Distribution of respondents by knowledge about diseases spread by drug use (*n* = 120).

Variables	Number (*n*)	Percentage (%)
Knowledge about diseases spread		
Yes	48	24.2
No	72	60.0

Diseases spread		
HIV/AIDS	21	17.5
Syphilis	8	6.7
Hepatitis B/C	7	5.8
Other blood-borne diseases	3	2.5
Don't know	81	67.5

Ever heard of (multiple answers possible)		
HIV/AIDS	68	56.7
Syphilis	21	17.5
Hepatitis B/C	45	37.5

Knowledge about protection		
Not injecting any more drugs	35	29.2
Cleaning syringe/needle with water	41	34.2
Using condoms	12	10.0
Taking medicines	6	5.0
No need	3	2.5
Don't know	23	19.2

Consequences of drug use (multiple answers possible)		
Affecting my health	103	85.8
Affecting my job/education	87	72.5
Affecting family relationships	96	80.0
Affecting socially	65	54.2
Others	51	42.5

**Table 5:** Sexual behavior of the respondents.

Variables	Number (*n*)	Percentage (%)
Sex with a regular partner		
Yes	76	63.3
No	19	15.8
No response	25	20.8

Sex in the last 3 months		
Yes	87	72.5
No	20	16.7
Not applicable	13	10.8

Used condom in the last sexual intercourse		
Yes	14	11.7
No	84	70.0
No response	22	18.3

Used condom with any partners		
Sometimes	19	15.8
Never	72	60.0
No response	29	24.2
